# Body condition and adrenal glucocorticoid activity affects metabolic marker and lipid profiles in captive female elephants in Thailand

**DOI:** 10.1371/journal.pone.0204965

**Published:** 2018-10-02

**Authors:** Treepradab Norkaew, Janine L. Brown, Pakkanut Bansiddhi, Chaleamchat Somgird, Chatchote Thitaram, Veerasak Punyapornwithaya, Khanittha Punturee, Preeyanat Vongchan, Nopphamas Somboon, Jaruwan Khonmee

**Affiliations:** 1 Graduate Program in Veterinary Science, Faculty of Veterinary Medicine, Chiang Mai University, Chiang Mai, Thailand; 2 Center of Excellence in Elephant and Wildlife Research, Faculty of Veterinary Medicine, Chiang Mai University, Chiang Mai, Thailand; 3 Center for Species Survival, Smithsonian Conservation Biology Institute, Front Royal, Virginia, United States of America; 4 Department of Companion Animal and Wildlife Clinic, Faculty of Veterinary Medicine, Chiang Mai University, Chiang Mai, Thailand; 5 Veterinary Public Health Centre for Asia Pacific, Faculty of Veterinary Medicine, Chiang Mai University, Chiang Mai, Thailand; 6 Department of Medical Technology, Faculty of Associated Medical Sciences, Chiang Mai University, Chiang Mai, Thailand; 7 Small Animal Teaching Hospital, Faculty of Veterinary Medicine, Chiang Mai University, Chiang Mai, Thailand; 8 Department of Veterinary Bioscience and Veterinary Public Health, Faculty of Veterinary Medicine, Chiang Mai University, Chiang Mai, Thailand; University of Tasmania, AUSTRALIA

## Abstract

Studies in western zoo elephants have found relationships between body condition and physiological function, and identified mitigating management strategies to optimize health and welfare. A similar methodological approach was used in this study, which evaluated a body condition score (BCS; 1 = thinnest, 5 = fattest) every other month and fecal glucocorticoid metabolite (FGM) concentrations twice monthly in 33 tourist camp elephants in Thailand for a 1-year period to assess seasonal variations, and determine how lipid profiles [total cholesterol (TC), low density lipoproteins (LDL), high density lipoproteins (HDL), triglycerides (TG)] and metabolic parameters [insulin, glucose, fructosamine, glucose to insulin ratio (G:I)] related to measures of body condition and adrenal function. The most prevalent BCS was 3–3.5 (60.6%), with 27.3% at BCS = 4 (overweight) and 12.1% at BCS = 4.5–5 (very overweight); no elephants had a BCS <2. BCSs were higher in rainy and winter seasons compared to summer, with FGM, TG, HDL, LDL, and insulin also higher in the rainy and/or winter seasons (p<0.05). By contrast, TC and glucose were lowest in the rainy season. FGM measures were negatively associated with two environmental factors: temperature and rainfall, but not humidity. Positive correlations were found between BCS and TC, LDL, and HDL, and between FGM and TC, HDL, glucose, and insulin (p<0.05), whereas BCS and FGM were both negatively associated with the G:I (p<0.05). However, there was no relationship between BCS and FGM among the camp elephants. Using BCS and FGM measures as outcome variables in separate regression models, this study found high BCS and elevated FGM concentrations were associated with altered lipid profiles and metabolic status in elephants. Furthermore, more work hours/day was associated with better body condition and health measures. Thus, being overweight and exposed to factors that increase adrenal activity could adversely affect health status, requiring alterations in management for some individuals, whereas exercise appears to have a protective effect.

## Introduction

Healthy populations of elephants are essential to long-term global conservation, especially in the face of declining numbers. Wild elephant numbers throughout Asia have declined at least 50% over the last three generations, and without changes to conservation management, some populations are bound to disappear within the next century [1, 2]. Captive propagation is one strategy to ensure species survival [3], especially when conducted in range countries [4]. However, many Asian elephant (*Elephas maximus*) populations *ex situ* are not self-sustaining due to poor reproduction [5] or health problems [6–8].

The elephant is a national symbol of Thailand, and an integral part of Thai and Buddhist culture, and national economics. In the early 1900's, there were ~100,000 captive elephants in Thailand, almost all used for logging [9], a practice that was banned in 1989. Today, there are ~3,500 captive elephants, mostly (95%) privately owned [10], that are used primarily for tourism. Poor sustainability of captive elephants in Thailand is a concern, and has resulted in increasing efforts to improve breeding and population management [4]. The majority of tourist elephants today are located in the north and northeast of the country (~60%), primarily in Chiang Mai province where this study was conducted. In species with slow-growth populations (i.e., animals that mature late, produce small numbers of offspring, and have long life spans), sustainability is influenced by factors such as adult survival [11–14] and reproduction [15]. Asian elephants are a long-lived species, and produce only a few calves in their lifetime, so it is important to better understand factors affecting health and reproduction to prevent further population declines.

There are a number of ways to evaluate animal health status, apart from routine physical examinations. One is a visual body condition score (BCS), which has become an indirect, effective, and inexpensive tool in veterinary practices for assessing body fat, especially as it relates to problems with obesity [16]. For Asian elephants, several BCS indexes have been developed using 5- (Morfeld et al.,2016 [17]), 10- (Wijeyamohan et al., 2015 [18]) and 11- (Wemmer et al., 2006 [19]) point scales. In many species, high body condition or obesity can have negative effects on lipid profiles, including elevated serum triglycerides (TG) [20, 21], total cholesterol (TC) [20, 21], low density lipoprotein (LDL) [22–24], and very low density lipoproteins (VLDL) [20, 25]. Abnormally elevated blood glucose, TG, and cholesterol have been linked to a number of disease problems, collectively called metabolic syndrome, such as hypertension, hyperlipidemia, insulin resistance, and type 2 diabetes [26, 27]. By contrast, few studies have examined these health factors in relation to body condition in elephants, and those were only on animals in western zoos. For example, negative associations were found between the BCS and glucose to insulin ratio (G:I) in zoo Asian and African elephants [28], and fat-free mass and circulating glucose in African elephants [29], whereas Morfeld and Brown [30] reported a positive correlation between BCS and insulin concentrations in African elephants. Subsequent studies demonstrated the protective effects of increased exercise and feeding diversity on BCS and metabolic function in zoo-housed elephants [28], which could have application to tourist elephants in Thailand that participate in varying types of physical activities. Another favorite activity of tourists is feeding bananas and sugar cane, leading to concerns about whether that is linked to obesity and metabolic problems.

In addition to obesity, stress can also affect metabolic health and lipid parameters. Glucocorticoids (GCs) are key regulators of whole-body homeostasis, and provide an organism with the capacity to resist environmental changes and invasion of foreign substances [31]. Glucocorticoids modulate a large number of physiological actions involved in metabolic, inflammatory, cardiovascular, and behavioral processes [32]. Mechanisms by which GCs orchestrate these effects include increasing hepatic glucose production [33], decreasing peripheral glucose uptake into muscle and adipose tissue [34, 35], increasing breakdown of fat and muscle to provide additional substrates for glucose production [36–38], and inhibiting insulin release from pancreatic beta cells [39]. While important for maintaining metabolic equilibrium, excessive GC exposure for prolonged periods can have devastating effects on health. Patients with hyper-GC production exhibit abdominal obesity, increased overall body fat, high blood pressure, insulin resistance, and a preference for fatty foods [40]. Glucocorticoids can affect fat metabolism by stimulating lipolysis, which leads to the release of free fatty acids into circulation [32]. Moreover, it has been shown that GCs play a role in the redistribution of body fat from periphery to visceral depots [41]. Working elephants in Thailand interact with the public in a variety of ways, including performing in shows, trekking, bathing, and painting. Often these activities are not closely monitored or regulated, and could be sources of stress to individual animals. Fecal GC metabolite analysis techniques have been developed for numerous species, which has led to improved *ex situ* management [42]. The ease of fecal collection without animal disturbance, and that data reflect pooled values over time that are less affected by pulsatile or diurnal changes in secretion makes this a particularly attractive approach for wildlife.

In recent years, the welfare of captive elephants has become a topic of intense debate among the lay public, government agencies, and the scientific community. Therefore, the aim of this study was to examine relationships between BCS and fecal GC metabolite (FGM) concentrations on metabolic function (insulin, glucose, fructosamine, G:I) and lipid profiles (TC, TG, HDL, LDL) in tourist camp elephants in Thailand. The hypothesis was that elephants with higher adrenal activity and BCSs will exhibit more metabolic derangements than those with normal BCS and lower FGM concentrations. The goal is to better understand what factors are related to health and welfare of working elephants in Thailand so that evidence-based standards can be developed to create healthy, self-sustaining populations.

## Materials and methods

### Environmental data

Weather in Thailand is hot and humid, with three official seasons: summer (16 February–15 May), rainy (16 May–15 October) and winter (16 October–15 February). Information on daily temperature (°C), amount of rainfall (mm/day), and humidity (%), averaged by month, was obtained from The Northern Meteorological Center, Meteorological Department, Ministry of Information and Communication Technology, Chiang Mai, Thailand (Thai Meteorological Department, 2016). A thermal–humidity index (THI) was calculated based on air temperature and relative humidity using the following formula: THI = (1.8×*T*db+32) − (0.55−0.0055×RH) × (1.8×*T*db−26), where the Tdb is the temperature of air measured by a thermometer freely exposed to the air, but shielded from radiation and moisture, and RH is the relative humidity (%) [43].

### Animals

This study was approved by the Faculty of Veterinary Medicine, Chiang Mai University, Animal Care and Use Committee (FVM-ACUC; permit number S39/2559). Thirty-three adult female Asian elephants (age range, 18–50; mean, 34.2±7.3 years) were housed at five tourist camps within 43–72 km of the Chiang Mai University Veterinary Faculty (latitude 18°47'N, longitude 98°59'E, altitude 330 m) (Table 1). At four of the camps, tourists interacted with elephants through riding programs (bareback or with a saddle) and feeding supplementary foods. The fifth camp offered no tourist activities other than observation of elephants in a large field, and being taken for a bath at a local river. Work hours per day was the time elephants were involved in tourist activities, like giving rides or walking to a river for bathing. For saddle and bareback riding, it equated to the number of rounds of riding per day times the minutes per riding round. Elephants were fed primarily corn stalk, *napier grass* (*Pennisetum purpureum*) and bana grass (*Pennisetum purpureum* X, *P*. *americanum* hybrid) with unlimited access to fresh water. Animals were given an annual physical examination by staff veterinarians, and were in good health during the study.

**Table 1:** Description of each elephant camp in the study (Camps A–E). Information includes number of years the camp has been in operation (camp age), total number of elephants in the camp, number of elephants participating in the study, participating elephant mean age (±SEM) and range, type of work with tourists, hours worked per day, and primary and supplemental food items.

Variable	Camp A	Camp B	Camp C	Camp D	Camp E
Camp age (years)	9	27	29	14	40
Total elephant number	46	66	52	68	76
Participating elephant number	6	6	6	11	4
Elephant age (years)	28.5±1.8 (22–34)	36.8±2.9 (23–43)	35.3±3.8 (20–45)	35.8±2.3 (25–50)	32.2±3.0 (22–40)
Type of work	Bareback riding	Saddle riding	Saddle riding	No riding	Saddle riding
Work time (hours/day)	1.3	2.3	3.8	0.2	3.3
Diet					
Primary	*Napier grass*, cornstalk	*Napier grass*, cornstalk	*Napier grass*, cornstalk	*Napier grass*, bana grass, cornstalk	*Napier grass*
Supplementary	*Bamboo*, *sugarcane*, *banana*	*Banana*, *sugarcane*	*Banana*, *sugarcane*	*Hay*, *banana*, *watermelon*, *pumpkin*, *cucumber*	*Bamboo*, *sugarcane*, *banana*

### Body condition scoring

Once every 2 months, rear and side view photographs were taken of each elephant to permit a visual evaluation of the backbone, rib bone and pelvic bone areas, and scored 1–5 (1 = thinnest; 5 = fattest) as described by Morfeld et al. [17], except that scoring was done in 0.5-point, rather than 1-point, increments. All photos were evaluated by three experienced elephant veterinarians, and the scores averaged. Intra-class correlations determined the inter-assessor reliability was 0.85.

### Blood collection

Blood samples (10 ml) were collected from each elephant from an ear vein by elephant camp staff or Chiang Mai University veterinarians twice monthly for 1 year. All elephants were conditioned to the blood sampling procedure. Blood was centrifuged at 1,500 x g for 10 minutes within a few hours of collection, and the serum stored at -20°C until processing and analysis.

### Metabolic markers analysis

Serum glucose was measured by a hexokinase method using an automated glucose analyzer (Glucinet T01-149, Bayer, Barcelona, Spain), with quinoneimine measured at 530 nm. Serum fructosamine was measured by a colorimetric method using nitrobluetetrazolium [44] in a Biosystems BA400 clinical chemistry analyzer (Biosystems S.A., Barcelona, Spain). A solid-phase, two-site bovine insulin enzyme immunoassay (EIA; Cat. No. 10-1113-01; Mercodia, Uppsala, Sweden), validated for elephants, was used to measure serum insulin concentrations [30]. Colorimetric responses were determined spectrophotometrically at 450 nm filter with an Opsys MR Microplate Reader (TECAN Sunrise^TM^ microplate reader; Salzburg, Austria). All samples were analyzed in duplicate; intra- and inter-assay CVs were <10% and <15%, respectively.

### Lipid profile analysis

Serum lipids were quantified using a Mindray BS Series analyzer (Mindray BS-380, Shenzhen Mindray Bio-Medical Electronics Co., Ltd.). Total cholesterol was measured by a cholesterol oxidase-peroxidase (CHOD-POD) method. Triglycerides were measured by a glycerokinase peroxidase-peroxidase (GPO-POD) method, with a sensitivity of 0.1 mmol/L (99.7% confidence). The lowest measurable concentration was 0.1 mmoL/L (99.7% confidence) for TC, and 0.05 mmol/L for both HDL and LDL.

### Fecal extraction and GC metabolite analysis

All chemicals were obtained from Sigma Chemical Company (St. Louis, MO), unless otherwise stated. The fecal extraction method was based on Brown et al. [45]. Briefly, wet fecal samples were dried in a conventional oven at 60°C for ~24–48 hours and stored at -20°C until extraction. Frozen dried fecal samples were thawed at room temperature (RT), mixed well and 0.1 g (±0.01) of dry powdered feces placed in a glass tube containing 90% ethanol in distilled water. Samples were extracted twice by boiling in a water bath (96°C) for 20 minutes and adding 100% ethanol as needed to keep from boiling dry. Samples were centrifuged at 1,500 x g for 20 min, and the combined supernatants dried under air in a 50°C water bath. Dried extracts were reconstituted by vortexing for 1 min in 3 ml ethanol, dried again, and then diluted and vortexed in methanol for analysis. Extracts were stored at –20°C until EIA analysis.

Concentrations of FGM were determined using a double-antibody enzyme immunoassay (EIA) with a polyclonal rabbit anti-corticosterone antibody (CJM006) that has been validated for Asian elephants [46]. Second antibody-coated plates were prepared by adding 150 μl of anti-rabbit IgG (0.01 mg/ml) to each well of a 96-well microtiter plate, and incubating at room temperature (RT) for 15–24 h. The wells were then emptied and blotted dry, followed by adding 250 μl blocking solution and incubating for 15–24 h at RT. After incubation, all wells were emptied, blotted and dried at RT (Sanpla Dry Keeper, Sanplatec Corp., Auto A-3, Japan) with loose desiccant in the bottom. After drying (humidity <20%), plates were heat-sealed in a foil bag with a 1g desiccant packet, and stored at 4°C until use.

Samples (50 μl), diluted 1:3 in assay buffer, or corticosterone standards (50 μl) were added to appropriate wells. Corticosterone-horseradish peroxidase (25 μl) was immediately added to each well except for non-specific binding wells, followed by 25 μl anti-corticosterone antibody, and incubated at RT for 1 h. Plates were then washed four times with wash buffer (1:20 dilution, 20X Wash Buffer Part No. X007; Arbor Assays, MI) and 100 μl of TMB substrate solution was added, followed by incubation for 45–60 min at RT without shaking. The absorbance was measured at 405 nm by a microplate reader (TECAN). Assay sensitivity (based on 90% binding) was 0.14 ng/ml. Samples were analyzed in duplicate; intra- and inter-assay CVs were <10% and <15%, respectively.

### Statistical analysis

Descriptive data were reported as mean ± standard error of the mean (SEM) and camp management variables were presented as a range or a frequency, depending on the type of data. Statistical analyses were performed using R version 3.4.0 (R Development Core Team, 2017). Repeated measures data were analyzed using Generalized Estimating Equations (GEE) to determine: 1) the effects of BCS and FGM on metabolic and lipid panel results; 2) seasonal and climate factor effects on metabolic and lipid function; and 3) relationships among metabolic and lipid panel measures. Differences in mean metabolic and lipid profiles between BCS groups and seasons were further analyzed by Tukey’s post-hoc tests after GEE analyses. Correlations between individual FGM and metabolic hormones or lipid measures in each elephant (n = 33) were analyzed using Pearson's tests for aggregated data. Mean monthly FGM were compared using GEE followed by a Tukey’s test. The significance level was set at α = 0.05.

## Results

**Fig 1:**
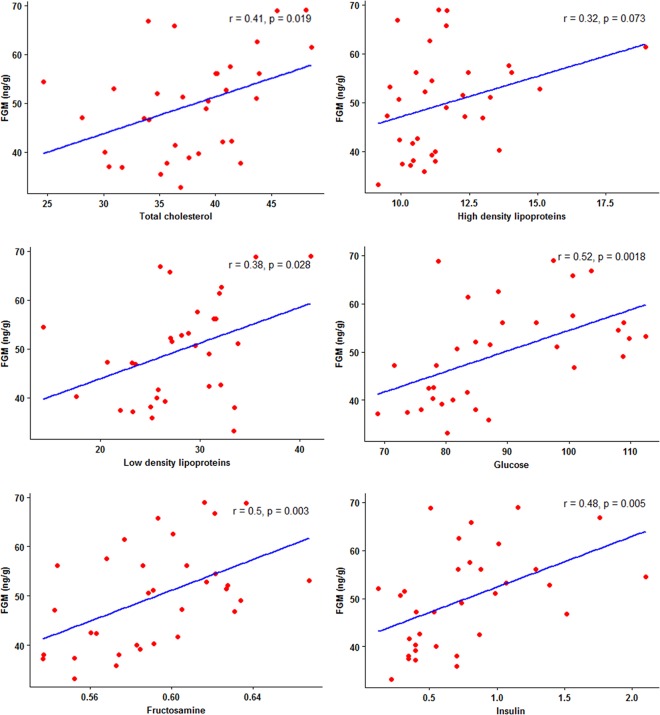
Relationships between fecal glucocorticoid metabolite (FGM) concentrations and metabolic and lipid measures. Pearson's correlation analyses illustrating relationships between FGM concentrations and total cholesterol, high density lipoproteins, low density lipoproteins, glucose, fructosamine, and insulin in female Asian elephants (n = 33) in Thailand.

Descriptive FGM, BCS, metabolic marker, and lipid profile measures are presented in Table 2, highlighting the variability in mean and range values across individuals. BCSs ranged from 2 to 5, with none scoring BCS = 1 or 1.5. Based on the yearly average of monthly mode values, numbers of elephants in each BCS category were: BCS = 2 (N = 1), BCS = 2.5 (N = 0), BCS = 3 (N = 14), BCS = 3.5 (N = 6), BCS = 4 (N = 7), BCS = 4.5 (N = 3), BCS = 5 (N = 2). Most elephants (42.4%) were a BCS = 3, with 33.3% scoring BSC = 4 and 5. Relationships between BCS and FGM on metabolic markers and lipid profiles are presented in Table 3. There were significant positive associations between BCS and TC, HDL and LDL. Fecal GC metabolite concentrations also were positively related to TC and HDL, as well as glucose and insulin. Both BCS and FGM were negatively correlated to G:I ratios. In separate Pearson's correlation analyses of individual means (n = 33), FGM levels were similarly correlated to TC, LDL, glucose, insulin, and fructosamine (p<0.05) (Fig 1). Work time (hours per day) was negatively correlated with FGM concentrations (r = -0.69, p<0.01), HDL (r = -0.51, p<0.01), glucose (r = -0.78, p<0.01), fructosamine (r = -0.59, p<0.01) and insulin (r = -0.59, p<0.01), whereas the G:I was positively associated with work time (r = 0.32, p<0.05).

**Table 2:** Descriptive statistics. Mean (±SEM) and range values for body condition scores (BCS), fecal glucocorticoid metabolite (FGM) concentrations, lipid panel measures and metabolic factors in female Asian elephants (n = 33) in Thailand.

Parameters	Mean	Min—Max	Mean range
BCS	3.50±0.02	2.00–5.00	2.37–5.00
FGM (ng/g)	50.80±0.89	11.42–194.17	34.51–72.26
TC (mg/dL)	37.40±0.32	10.00–109.00	24.38–48.52
TG (mg/dL)	28.60±0.64	4.00–157.00	18.09–42.96
HDL (mg/dL)	11.60±0.10	2.00–26.00	9.17–18.96
LDL (mg/dL)	27.80±0.29	8.00–107.00	14.30–41.14
Glucose (mg/dL)	88.90±0.75	50.00–180.00	68.91–112.50
Fructosamine (mM)	0.59±0.01	0.38–0.92	0.54–0.67
Insulin (μg/L)	0.75±0.03	0.02–5.91	0.12–2.10
G:I	196.00±6.72	22.67–721.56	83.87–532.32

TC = total cholesterol; TG = triglycerides; HDL = high density lipoproteins; LDL = low density lipoproteins; G:I = glucose to insulin ratio.

**Table 3:** General Estimation Equation analyses. Relationships between health factors and body condition and adrenal steroid activity in female Asian elephants (n = 33) in Thailand.

Parameters	BCS			FGM		
	Intercept	Beta	P value	Intercept	beta	P value
TC (mg/dL)	2.742	0.021	0.001	38.370	0.330	0.009
TG (mg/dL)	3.428	0.004	0.210	50.742	0.005	0.920
HDL (mg/dL)	2.751	0.067	0.001	34.160	1.445	<0.001
LDL (mg/dL)	2.960	0.021	0.002	44.933	0.223	0.076
Glucose (mg/dL)	3.361	0.002	0.430	21.314	0.336	<0.001
Fructosamine (mM)	3.179	0.610	0.570	39.270	19.81	0.230
Insulin (ng/ml)	3.654	0.062	0.570	46.610	6.160	0.004
G:I	3.913	-0.001	0.009	54.248	-0.014	0.038

BCS = body condition score; FGM = fecal glucocorticoid metabolites; TC = total cholesterol; TG = triglycerides; HDL = high density lipoproteins; LDL = low density lipoproteins; G:I = glucose to insulin ratio.

Differences in FGM, metabolic marker and lipid profile measures related to BCS are shown in Table 4. Because of limited numbers, elephants were grouped into three BCS classes: 2.0–3.0 (N = 15); 3.5–4.0 (N = 13); and 4.5–5.0 (N = 5) for further analysis. Higher levels of TC and LDL were found in elephants with a BCS of ≥3.5 (overweight/very overweight) compared to those <3.5 (p<0.05). Elephants with BCSs of 4.5–5.0 had higher HDL, glucose and insulin levels, and the lowest G:I ratio (p<0.05). Triglyceride concentrations in the intermediate BCS group (BCS = 3.5–4) were higher than those in BCS = 2–3, but similar to BCS = 4.5–5. By contrast, FGM concentrations were similar across the BCS groups.

**Table 4:** Body condition effects on adrenal and health makers in elephants. Effect of body condition on mean (±SEM) and range values fecal glucocorticoid metabolite (FGM) concentrations, lipid panel measures and metabolic factors in Asian elephants in Thailand (n = 33).

BCS	FGM (ng/g)	TC (mg/dL)	TG (mg/dL)	HDL (mg/dL)	LDL (mg/dL)	GLU (mg/dL)	FRUC (mM)	INS (ng/ml)	GI
2.0–3.0	58.01±4.02 (20.00–194.00)	35.20±0.88^a^ (22.00–52.00)	24.70±1.97^a^ (7.00–85.00)	11.50±0.27^a^ (7.00–17.00)	25.00±0.97^a^ (9.00–43.00)	96.50±3.09^a^ (56.00–180.00)	0.59±0.01^ab^ (0.45–0.80)	0.91±0.12^a^ (0.04–5.91)	165.00±34.60^ab^ (22.70–483.00)
3.5–4.0	58.00±4.28 (15.70–159.00)	38.60±1.10^b^ (22.00–60.00)	34.50±2.99^b^ (8.00–110.00)	11.60±0.32^ab^ (9.00–21.00)	29.40±0.98^b^ (13.00–52.00)	92.10±3.50^a^ (52.00–152.00)	0.58±0.01^a^ (0.48–0.70)	0.91±0.14^a^ (0.07–2.92)	183.00±24.10^b^ (38.20–543.00)
4.5–5.0	60.93±8.72 (32.00–126.00)	40.60±2.03^b^ (28.00–55.00)	29.90±2.84^ab^ (10.00–59.00)	13.20±0.67^b^ (9.00–21.00)	29.80±1.23^b^ (21.00–38.00)	104.80±5.65^b^ (58.00–144.00)	0.61±0.01^b^ (0.57–0.67)	1.23±0.22^b^ (0.07–2.58)	85.00±10.20^a^ (40.70–142.00)

^a,b,c^Values for each variable differ among BCS categories within columns are significantly different (p<0.05).

BCS = body condition score; TC = total cholesterol; TG = triglycerides; HDL = high density lipoproteins; LDL = low density lipoproteins; GLU = glucose; FRUC = fructosamine; INS = insulin; G:I = glucose to insulin ratio.

**Fig 2:**
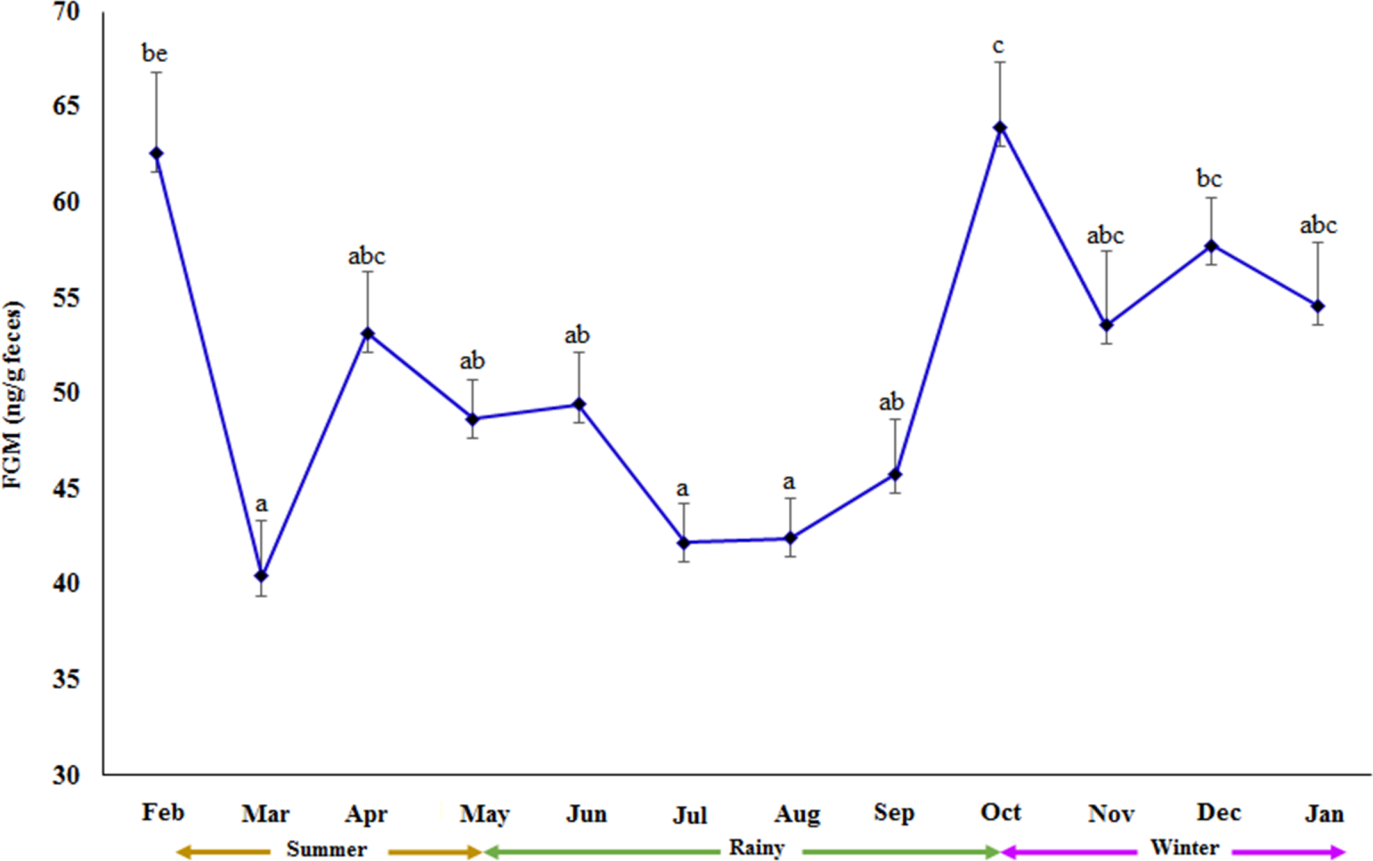
Seasonal pattern of fecal glucocorticoid metabolite (FGM) concentrations. Mean (± SEM) monthly fecal glucocorticoid metabolite (FGM) concentrations in female Asian elephants (n = 33) across the summer, rainy and winter seasons in Thailand. Superscripts designate significant differences in monthly mean FGM concentrations (p<0.05).

Seasonal effects on measured parameters are summarized in Table 5. All but the G:I was significantly affected by season. BCSs were 9–13% higher in elephants during the rainy and winter seasons compared to the summer, with TG, LDL, and insulin being higher in the winter months only. Mean FGM concentrations were ~28% higher in winter compared to the summer and rainy seasons. LDL was also higher in the rainy and winter seasons compared to the summer. By contrast, HDL was highest in the summer, intermediate in the winter and lowest in the rainy season, whereas glucose was high in the winter and summer seasons. The effect of season on longitudinal FGM concentrations is presented in Fig 2, with lower concentrations observed between March and September, representing mid-summer to rainy seasons. Relationships between environmental factors and BCS and FGM concentrations are presented in Table 6, with significant correlations noted between BCS and humidity, and between FGM and all environmental factors but humidity. There were significant negative effects of monthly temperature, rainfall, and THI on FGM measures.

**Table 5:** Seasonal effects on body condition, adrenal steroid hormones and health factors in elephants. Mean (±SEM) and range values in body condition scores (BCS), fecal glucocorticoid metabolite (FGM) concentrations, lipid panel measures and metabolic factors across the summer, rainy and winter seasons of female Asian elephants (n = 33) in Thailand.

Parameters	Summer	Rainy	Winter
BCS	3.20±0.13^a^ (3.00–4.00)	3.48±0.08^ab^ (2.00–5.00)	3.62±0.08^b^ (2.00–5.00)
FGM (ng/g)	47.40±1.79^a^ (11.42–147.00)	47.3±1.15^a^ (11.90–132.38)	60.00±1.95^b^ (15.45–194.17)
TC (mg/dL)	38.70±0.88^b^ (20.00–109.00)	36.00±0.45^a^ (10.00–77.00)	38.20±0.46^b^ (22.00–60.00)
TG (mg/dL)	25.10±1.70^a^ (4.00–157.00)	29.20±0.86^ab^ (6.00–88.00)	30.40±1.10^b^ (7.00–110.00)
HDL (mg/dL)	12.50±0.25^c^ (2.00–26.00)	11.10±0.13^a^ (5.00–26.00)	11.70±0.16^b^ (7.00–21.00)
LDL (mg/dL)	25.80±0.57^a^ (12.00–89.00)	27.70±0.56^b^ (8.00–107.00)	29.40±0.46^b^ (9.00–67.00)
Glucose (mg/dL)	92.90±1.13^b^ (65.00–160.00)	83.60±1.13^a^ (50.00–180.00)	92.90±1.56^b^ (52.00–172.00)
Fructosamine (mM)	0.60±0.004^b^ (0.48–0.77)	0.59±0.004^ab^ (0.38–0.92)	0.58±0.003^a^ (0.45–0.77)
Insulin (ng/ml)	0.76±0.08^ab^ (0.02–3.37)	0.59±0.05^a^ (0.03–2.67)	0.94±0.09^b^ (0.03–5.91)
G:I	207.00±19.60 (34.41–721.56)	188.00±11.90 (38.17–713.17)	163.00±12.40 (22.67–612.53)

^a,b,c^Seasonal mean values are significantly different across each row (p<0.05).

TC = total cholesterol; TG = triglycerides; HDL = high density lipoproteins; LDL = low density lipoproteins; G:I = glucose to insulin ratio

Summer: 16 February–15 May, rainy: 16 May–15 October, winter: 16 October–15 February

**Table 6:** General Estimation Equation analysis of seasonal relationships. Relationships between body condition scores (BCS) and fecal glucocorticoid metabolite (FGM) concentrations and environmental factors in female Asian elephants (n = 33) in Thailand.

Parameters	BCS			FGM		
	Intercept	Beta	P value	Intercept	Beta	P value
Temperature (°C)	4.238	-0.026	0.23	84.43	-1.211	0.001
Rainfall (mm)	3.310	0.041	0.057	53.65	-0.77	0.028
Humidity (%)	2.307	0.005	0.001	54.21	-0.043	0.600
THI	3.781	-0.003	0.835	103.93	-0.682	0.022

THI = temperature-humidity index

Several correlations were noted amongst the metabolic and lipid factors as shown in Table 7. Strong positive associations (p<0.001) included TC with TG, HDL with LDL, HDL with LDL and insulin, and glucose with fructose and insulin. By contrast, negative relationships (p<0.01) were found between the G:I ratio and HDL, glucose, and insulin.

**Table 7:** Relationships among metabolic and lipid factors. Correlation matrix presenting relationships between lipid panel measures and metabolic factors in female Asian elephants (n = 33) in Thailand.

	TC							
TG		TG						
HDL			HDL					
LDL				LDL				
GLU					GLU			
FRUC						FRUC		
INS							INS	
G:I								G:I

***p<0.001 (dark color),

**p<0.01 (medium color),

*p<0.05 (light color)

Blue = positive correlation. Red = negative correlation. TC = total cholesterol; TG = triglycerides; HDL = high density lipoproteins; LDL = low density lipoproteins; GLU = glucose; FRUC = fructosamine; INS = insulin; G:I = glucose to insulin ratio.

## Discussion

This was the first study to evaluate relationships between BCS and metabolic factors in Asian elephants in any range country, and the first to assess lipid profiles in relation to body condition in this species. We also present new evidence for a relationship between nutritional status and stress hormone levels in elephants, as indicated by associations between FGM concentrations and several measures of metabolic and lipid function. The most prevalent (mode) BCS in Thai elephants was 3–3.5 (60.6%), which is considered ideal [17]. Deviations from that were mostly in the higher BSC categories, with 27.3% at BCS = 4 (overweight) and 12.1% at BCS = 4.5–5 (very overweight); no elephants had a BCS <2. Overall, the working elephants in our study had better body condition than those in western zoos. For example, in the U.S. only 16.5% had a BCS = 3; 27% were BCS = 4 and 48% were BCS = 5 [17], whereas 75% were scored as overweight or very overweight in the U.K. [47]. This could be due to higher amounts of exercise, with tourist elephants engaged in many activities, including trekking, bathing, shows or walking with tourists [48], so inactivity is less of a concern. In the U.S., elephants that walked more than 14 hours/week had a decreased risk of BCS = 4 or 5 [17, 28]. Feeding diversity (i.e., presenting food in multiple ways) also was related to lower BCS in U.S. studies [17, 28]. Comparatively, there is little diversity in how tourist camp elephants are fed, which are given fodder throughout the day, even during trekking. Thus, increased exercise during tourist activities likely helps those elephants maintain better body condition, and in this study, elephants that worked more hours per day in the form of saddle or bareback riding had lower BCSs.

Despite the better overall body condition of tourist compared to western zoo elephants, over a third had scores that suggested they were overweight, and these exhibited alterations in lipid and sugar metabolism. A popular activity for tourists is feeding elephants, particularly bananas and sugar cane, which possess high concentrations of sucrose and other soluble sugars that could contribute to weight problems. Blood glucose values in this study agreed with non-fasted levels found in a previous study of Thai elephants [49]. Another finding was that very overweight elephants (BCS = 4.5–5) had higher levels of insulin. Insulin plays a central role in the regulation of blood glucose and energy homeostasis; however, high levels are associated with hypertension, obesity, dyslipidemia, and glucose intolerance in humans [50, 51]. It was not possible to fast elephants before blood sample collection in this study, and they had access to forage overnight, so we also evaluated the G:I ratio, which has been used for detection of insulin sensitivity in women [52], with lower values reflecting metabolic abnormalities. In the present study, BCS was predictive of G:I, and elephants with higher scores (BCS = 4–5) exhibited significantly lower G:I, a finding similar to U.S. studies [17, 28]. Comparatively, the overall G:I average value for the tourist elephants (G:I = 165) was slightly better than that in the U.S. (G:I = 110), although the ranges were similar (23–483 versus 14–430, respectively). In addition to glucose and insulin, serum fructosamine also was measured, which reflects glucose levels over the previous 2–3 weeks, and has been used to monitor and control blood sugar levels in diabetic patients [53], dogs and cats [54]. It has been reported to have positive correlations with body weight [55], body mass index [56] and waist circumference [57] in other species. This is the first report of fructosamine levels in Asian elephants, which were positively correlated with BCS and glucose, and so could be an additional marker of sugar homeostasis. Glycated hemoglobin (HbA1c) is another measure that provides average blood glucose levels over longer, 1–4 month periods, and is more common for monitoring diabetes in humans, although fructosamine tests are better for people with sickle cell or other blood disorders that can affect HbA1c levels [58]. We attempted to measure HbA1c in elephants by two ways: high performance liquid chromatography and a HbA1c-DIRECT EIA (BioSystems S.A., Costa Brava, Barcelona, Spain); however, levels were undetectable.

There was a seasonality in BCSs, with the average being lowest in the summer. Other studies have shown a shift towards thinner body condition during dry as compared to wet seasons in free-ranging Asian elephants [59]. The only climate factor related to BCS was a positive relationship with humidity, although the effect of rainfall approached significance. A variety of climatic drivers of body size declines have been proposed, and in ungulates, an indirect link between climate, resources and ungulate body mass has been established, and may be influenced by climate change [60, 61]. Warm springs reduce both relative winter mass loss and summer mass gain in adult bighorn sheep (*Ovis canadensis*), likely due in part to growth rate of plants and duration of access to high-quality forage [62]. A seasonal shift in the diet of southeast Asian elephants from high-quality grasses in the wet season to poorer quality grass during the dry season [63–65] could explain the lower BCS during the summer season. Although our study animals were not free-ranging, most fodder was sourced locally, and so would have been subject to seasonal influences. Quantifying the amount and frequency of food provided was beyond the scope of this study, but will be a focus of future studies to identify relationships between nutrient intake, body condition and health.

Serum TG concentrations were within the range reported for Asian elephants [17, 66], and lower for females with BCS = 2–3, like that of Morfeld et al. [17]. Significant relationships between BCS and other lipids were observed, with thinner elephants (BCS = 2–3) exhibiting lower TC, HDL, and LDL concentrations than those with higher scores. In humans, a number of metabolic changes are associated with obesity, high BMI or poor eating habits, including elevated TG, TC and LDL levels. Similarly, in dogs, increased plasma TC and TG concentrations are observed in association with obesity [20], and in horses, TG concentration is correlated with body condition scores [67, 68]. Dyslipidemia resulting from poor diets and inadequate exercise constitutes a major risk for development of heart disease and other health problems in women, and is characterized by increased levels of TC, LDL cholesterol and TG, and decreased levels of HDL [69–71]. Hyperlipidemia could pose problems for elephants, although incidence of cardiovascular disease appears to be relatively low [6]. There was variability in the types and duration of work experienced by elephants across the five camps, with regression analyses indicating significant correlations between work hours and lipid panel results, being negative for HDL, glucose, fructosamine and insulin, and positive for G:I. Moderate amounts of exercise have a positive impact on lipid levels in other species [72, 73]; thus, exercise may provide a protective effect on lipid and sugar homeostasis in elephants, and should be encouraged.

There was a positive association between FGM and glucose, fructose, insulin, and a negative correlation with G:I ratio, indicating a possible relationship between adrenal function and metabolism, as has been demonstrated in other species [74–76]. Elevated and sustained cortisol secretion during chronic stress can lead to central obesity, hypertension, glucose intolerance, and dyslipidemia in humans [77]. Administration of exogenous GCs also can increase plasma insulin and TG levels in women, whereas adrenalectomy reduces plasma insulin, glucose and TG levels [78]. Studies to understand how management factors affect stress responses in captive animals are key to improving welfare [79], and are beginning to be applied to elephants in westerns zoos [47, 80], which will be used as models for subsequent studies of working tourist elephants in Asia.

Last, there was an effect of season on FGM, with higher concentrations during the winter when temperatures and rainfall were lower. Saliva cortisol in zoo Asian elephants in Spain were highest between October and December [81], in agreement with our results. The need for more energy to maintain optimum body temperature and ensure survival in cooler temperatures could be related to this finding. Although the climate in Thailand is relatively warm, elephants have been known to shiver on cool winter days (personal observations). In other ungulates, higher GC levels during winter have been found in white-tailed deer (*Odocoileus virginianus*) [82] and mule deer (*Odocoileus hemionus*) [83]. Elevated circulating GC levels as a response to cold stress also were documented in reindeer (*Rangifer tarandus*) [84] and in farm animals [85]. In our study, FGM concentrations were influenced by several environmental factors, and negatively associated with temperature, rainfall and THI. Interestingly, this pattern differed significantly from that of logging elephants in Myanmar, where FGM levels were positively correlated with rainfall, but not with temperature [59]. In that study, FGM concentrations were highest in June, July and August, which corresponded to the end of the hot season and start of the monsoon and work season [59]. In that population, the shift from rest to intense work periods appears to have resulted in a marked increase in adrenal activity. Increased workloads have been associated with increased adrenal corticoid activity in horses and humans [86, 87]. However, the negative relationship between FGM and work hours in this study suggests it may not only be work hours that are affecting adrenal activity in Thai elephants, but perhaps other factors like a greater number of tourists taking advantage of the nicer climate in the winter, all of which should be explored more fully.

## Conclusions

Using BCS and FGM as outcome variables in regression models, high BCS and FGM were predictors of higher and potentially unhealthy metabolic and lipid levels in female Asian elephants. This study provides the first evidence that altered metabolic marker and lipid levels are associated with high BCS and adrenal steroid hormone measures in tourist camp elephants in Thailand, and that problems may be exacerbated during the high tourist season (winter and rainy seasons). Future studies will focus on what factors specifically affect elephant health and well-being, and the potential benefits of limiting the amount of high calorie treats (bananas, sugar cane) given to elephants by tourists, ensuring animals receive appropriate levels of exercise to reduce fat and increase muscle mass, and reducing stress by limiting workloads and numbers of tourists interacting with individual elephants, especially during the high season.
